# Characterization of Full Bridge Strain Transducers for Haulage Equipment Payload Distribution Monitoring

**DOI:** 10.3390/s26082374

**Published:** 2026-04-12

**Authors:** Jean-Pierre Strydom, Steve Schafrik, Zach Agioutantis, Matt Beck, Joseph Sottile

**Affiliations:** 1 Department of Mining Engineering, University of Kentucky, Lexington, KY 40504, USA; jst414@uky.edu (J.-P.S.); steven.schafrik@uky.edu (S.S.); joseph.sottile@uky.edu (J.S.); 2 Department of Materials Engineering, University of Kentucky, Lexington, KY 40504, USA; m.beck@uky.edu

**Keywords:** force-shunt strain transducer, transient strain, strain gage, shuttle car payload

## Abstract

Creating a dependable approach for identifying both the mass of a shuttle car and how material is distributed in it removes the need for equipment operators to manually engage the flight chain. The quantification of environmental and installation conditions and the extent of influence considering their combined contribution towards inaccurate or exclusive measurements are to that degree limited. This experimental study investigated how two different strain transducers—installed in a force-shunt configuration—respond to thermo-mechanical loads when used to determine load distribution and position. Initial observations indicated that thermal effects at the installation site contributed to measurement inaccuracies or exclusive readings. The investigation quantified the impact of environmental and installation variables on measurement accuracy and found this influence to be indirectly linked to the mechanical properties of the substrate to which the strain transducers were mounted. Mounting bolt torque was determined to exert a negligible effect on strain measurement accuracy for the custom-built strain transducers. Nonetheless, both transducers failed to consistently return to the selected baseline at the start of experiments since thermal dependence persisted at the balanced state following the first cycle of loading. The research indicated that the custom-built force-shunt strain transducers are an effective means for mapping the profile and location of coal in shuttle cars, provided that the systems are subjected to continuous and cyclic rebalancing to maintain accuracy.

## 1. Introduction

For well over a decade, the mining sector has continued to face continuous interruptions to operations due to accidents stemming from powered haulage and machinery; with MSHA’s 2025 Mine Injury and Worktime quarterly reports indicating a 20% average accident rate for all operator injuries [1]. These risks can be mitigated by reducing the manual intervention needed for machinery operation, such as the loading of shuttle cars, where operators currently manually adjust conveyor chains to achieve an even load distribution. Automating this task requires precise data on the loaded coal location, which is ideally gathered by measuring the force applied to the load bed; however, while force-flow transducers supersede force-shunt transducers in measurement accuracy—provided that the absolute force to be measured tides through the transducer itself—this mechanism imposes significant design and capacity constraints when applied to large payloads distributed across the length of a shuttle car. Consequently, because a direct scale requires all induced forces to be channeled through the measurement device, these structural limitations motivate the exploration of indirect approaches to quantify deformation as a more practical method for monitoring load profiles. This practical hurdle is much like trying to weigh a heavy shipping container by placing a single scale under one corner; to get a true reading, the entire weight must be perfectly balanced on the scale, which is often physically impossible for distributed mechanical loads.

**Figure 1:**
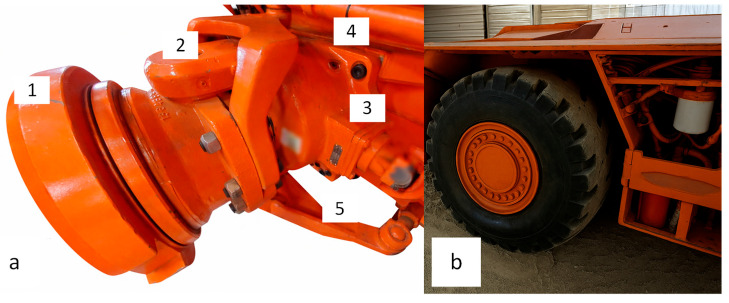
Possible strain transducer installation locations. (**a**) Viable installation points identified on shuttle car wheel hub. (**b**) Shuttle car wheel and front view of wheel hub assembly.

The application requires payload monitoring that favors accuracy to quantify both the coal load as well as the distribution profile within a shuttle car load bed. The symmetric geometry of the coal load bed, combined with space constraints that limit easy access for calibration and maintenance of strain transducers, determined the location of the installation position. As illustrated in Figure 1, the shuttle car wheel hubs were identified as the ideal positions to satisfy these criteria. Nevertheless, the confined space in which these haulage vehicles operate also ensures that mounting on the suspension often remains less practical; instead, variations of a pivoting axle supporting the machine’s discharge end wheels and fixed axles on the loading end are predominantly used [2]. Since wheel units were designed to limit chassis height and remain rigid, durable, and robust, these infrangible cast pivot axles have been deployed in most underground coal mines. This assertion is supported by the fact that independent suspension has remained an optional amendment to the standard machine specification [3,4]. Therefore, guaranteeing minimal complexity and maintenance whilst the shuttle car remains loaded at low speeds in restricted entries requires other means that do not involve linear displacement measurements to monitor payloads.

**Figure 2:**
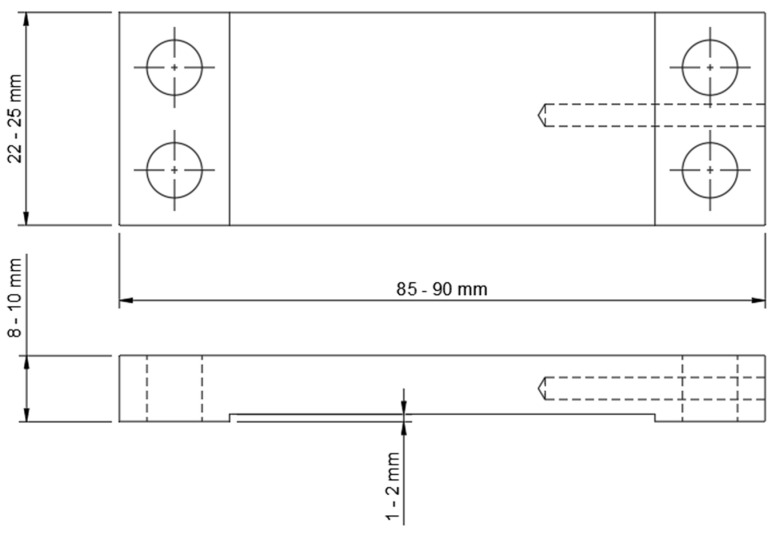
Overall dimensions of the strain transducer, modified from [5].

**Figure 3:**
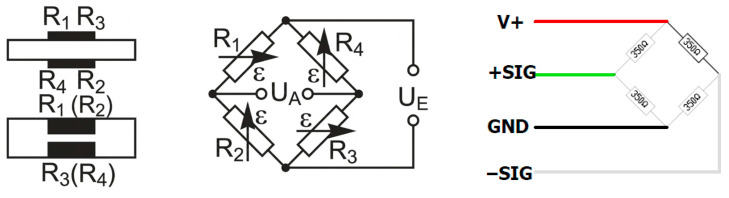
Custom-built strain transducer—strain gage arrangement and wiring diagram. U_A_ represents the output or differential voltage signal (−SIG to +SIG); U_E_ illustrates excitation voltage (GND to V+).

This experimental investigation primarily aimed to quantify the extent to which thermal dependence affects the accuracy of strain measurements by exposing four identical custom-built strain transducers (Figure 2 and Figure 3) and two commercially produced HBK SLB 700A full Wheatstone-bridge strain transducers to expected field conditions. The two additional transducers were built to verify consistent behavior and enable identification of possible design flaws that are considered to be mitigated by the commercially manufactured product.

The operating conditions, however, included controlled temperature fluctuations while external loads were applied via a shunt, by vibration, and varying mounting-torque levels. Additionally, the difference between force-flow and force-shunt load-transfer mechanisms was illustrated with 4-point bend testing. This investigation, therefore, focused on conditions typical of shuttle cars operating in underground room-and-pillar mining environments, and the findings establish a benchmark for the thermo-mechanical behavior of load cells under force shunt, providing a reference framework for informed sensor selection. Accordingly, the practicality of circumventing anomalies identified for a specific environmental setting could consequently be discussed.

## 2. Background

In ideal conditions, the strain detected by a strain transducer should replicate the deformation of the mounted object with complete fidelity. Prior studies indicate that the precision of strain measurements is significantly affected by installation factors, particularly the relative stiffness between the transducer and the local stiffness of the mounting surface [6]. Lin et al. [7] also noted that temperature-induced drift causes the indicated strain to deviate from its balanced state upon removal of the mechanical load. This effect becomes more pronounced over time. The results further demonstrated that apparent strain arising from abrupt mechanical changes can be eliminated by subtracting mechanical strain change per unit time from the measured signal. Likewise, ref. [8] noted that the zero-point shifts caused by thermal variations can be mitigated by incorporating temperature-compensating resistors within the input terminals and Wheatstone bridge arms. However, this approach becomes less effective when thermal gradients develop across the transducer. Despite these insights, literature offers limited quantification of how installation and environmental attributes collectively contribute to measurement inaccuracies or exclusivity.

The specific operational needs for a shuttle car include handling dynamic loads across varied payloads, rapid installation on accessible surfaces, and durability against environmental factors such as moisture and dust. Consequently, force-shunt strain transducers were selected over high-precision force-flow transducers for monitoring loads, because they enable simplified on-site mounting and reduce installation time. The use of these strain transducers provides a robust alternative to strain gages in ever-changing and inconsistent environments. These devices, frequently called load cells, use an indirect method to measure strain by detecting the deformation of a transducer or deformable object intended to follow the structural element’s movement. Load cells, however, are calibrated force transducers with an output signal directly proportional to the applied force, typically expressed in mV/V at full-scale force [9,10]. Strain transducers, on the other hand, operate on a similar principle, but they are not rated for force sensitivity; rather, they measure change in resistance linearly scaled from change in deformation over a single strain gage or Wheatstone bridge circuit.

The method for detecting deformation allows quantification of local strain without the need to direct the entire applied load through the transducer itself [10]. As opposed to force-flow strain transducers, the strain transducers in force shunt generally consist of strain gages bonded to host elements that add local stiffness to the underlying component. These transducers typically incorporate four electrical strain gages configured as a full Wheatstone bridge to convert mechanical energy into proportional electrical signals. The accuracy of these measurements remains dependent on the ratio between the transducer’s stiffness and the local stiffness of the host element. By strengthening the host element’s resistance to stress, the system enhances the resulting electrical output. This mechanical advantage subsequently refines the sensitivity required to identify minor changes in deformation across the structural body.

Data collected from the deployment of four custom-built strain transducers on a shuttle car demonstrated measurement responses that were both inconsistent and lacked repeatability under applied stress. Initial findings suggested that the primary cause of this data variability was the environmental thermal load present at the specific installation position. Technical literature indicates that such instabilities may be linked to the properties of the material to which the gages are bonded, the thermal coefficient of resistance (TCR) of the strain gages, and the influence of thermal hysteresis.

Ref. [11] demonstrated that, within operating temperatures ranging from 0 to 40 °C, despite a constant gage resistance, even slight changes in the TCR can lead to notable zero-point thermal shifts. Conversely, ref. [12] found that temperature fluctuations from −10 to 40 °C do not substantially affect the hysteresis of these sensors. Regarding TCR and the use of compensating resistors, ref. [13] demonstrated that for a precision load cell effectively compensated to reduce data variance, the change in sensitivity and the output at zero force can both be successfully restricted to less than 0.001% per °C. Consequently, to determine definitively whether the inconsistent and unrepeatable data are directly attributable to the specific thermal conditions at the installation point, an application-specific experimental analysis is required.

Ref. [14] identifies 10 environmental influences and external factors that have a substantial impact on thermal dependence, which is evident at the balanced condition: a strain gage’s previous exposure to thermo-mechanical cycling, the condition of the test article itself, electrical connections within the Wheatstone bridge circuit (particularly when strain gages are connected with one another or to the amplifiers using long cables), strain gage mounting, mechanical loading, the electrical resistance of strain gages, temperature, elasticity of the material (which includes the elements to which strain gages are bonded directly and indirectly), humidity, and the protection interference from the power source. These phenomena are well documented in the literature, and numerous investigations have attempted both to demonstrate and to mitigate their effects.

For instance, Yi [11] proposed a computational approach that incorporates the influence of an auxiliary resistor when calibrating the zero point to the desired condition. Similarly, Abhiram [15] applied a linear regression—temperature-based—compensation method to improve load cell precision, though the study ultimately concluded that more sophisticated machine-learning methods are needed to further reduce thermal drift. Richards [16] found that the temperature difference between strain gages and the material to which it is bonded is significant, and subsequently found a correlation for strain gage measurements involving transient temperature changes at heating rates exceeding 5.6 °C/s. Johnson [17] supports this assertion and explains that the so-called dummy gage technique is ineffective when the transient heating or dynamic heating rates occur, since it is improbable to achieve the same temperature on both the active and passive gages simultaneously. Johnson [17] further explains that self-compensated strain gages do not address zero output over the entire temperature range, and recommends that a thermocouple or temperature sensor be placed on top of the test article, next to the strain gage, and states further that this procedure is ineffective for heating rates that are large or exceed 5.6 °C/s.

Utilizing both numerical simulations and practical verification, Zhuang et al. [18] introduced a placement strategy for temperature-compensation resistors (nickel sheets) designed to offset the temperature-dependent field effects present in strain-gage load cells. These nickel-based components are specifically integrated to counteract span shifts, although the variation of the TCR is identified as the primary driver of zero-point instability. Accordingly, researchers have demonstrated that thermal gradients can induce internal forces that act upon the elastic element like an external load after subjecting a series of load cells to temperature conditions not within their compensated range. These environmental factors led Mattingly et al. [19] to observe a considerable variation between various load cells from the same manufacturer and model. Nonetheless, Cazzani et al. [20] classify the various approaches in four categories: analytical techniques, strain gage characterization in climate-controlled environments, installation of a passive gage(s) near active gage(s), and incorporation of self-compensated gages designed for specific materials applications.

Compensation for thermal expansion itself is inherently constrained by the physical conditions of the strain gages themselves: the bridge circuitry (electrical connections within the bridge circuit) and substrate to which strain gages are bonded. As per Hoffmann [14], these interference effects can be compensated for with 95 to 98% accuracy without the interplay of parasitic effects induced by the environment in which the strain gages are deployed. The simplest approach involves setting the strain gage circuit to zero at the test temperature and then preventing subsequent temperature change during the measurement. No thermal or mechanical induced stress should be present before zeroing the circuit. Naturally, this process is not always practical. Where the temperature compensation of the bonded strain gages is insufficient to address parasitic effects or where a strain gage is not available to match the thermal expansion coefficient of the test object’s material, the Wheatstone bridge is used [14,21]. However, to inform the temperature-compensation strategy, the mechanisms and behavior of the strain transducer’s response to temperature should be identified.

## 3. Methods, Materials and Simulated Environmental Conditions

The dominant environmental influences that distort strain measurements are inherently tied to the operating environment and cannot be practically eliminated. Consequently, only transducers that demonstrate minimal sensitivity or maintain consistent performance under such conditions are suitable for application. To replicate the anticipated parasitic effects while simultaneously applying mechanical loading, the study examined a set of environmental and installation parameters defined within the design specifications of HBK’s SLB-700A strain transducer [5]. This transducer was selected primarily owing to its ability to measure strain in a defined direction up to the maximum expected temperature of the shuttle car wheel housing while in operation. Specifically, the evaluation of the HBK SLB-700A considered operating temperatures ranging from 35 °C to 60 °C, mounting-torque levels between 8 N·m and 16 N·m (product design constraint), and vibration frequencies within its specification of 10 Hz to 500 Hz.

The characterization of the strain transducers continued until a consistent or accurate response from each transducer is evident. Yet transducers were subjected to induced temperature loads in conjunction with mechanical loads, for both uniform and non-uniform gradients, for a minimum of three replications. To address systematic errors and eliminate confounders, randomization was achieved by swapping the input circuit connections between two respective strain recorders over multiple days without adjusting for ambient air temperature changes. Induced load was reset at the end of cooling cycles.

**Figure 4:**
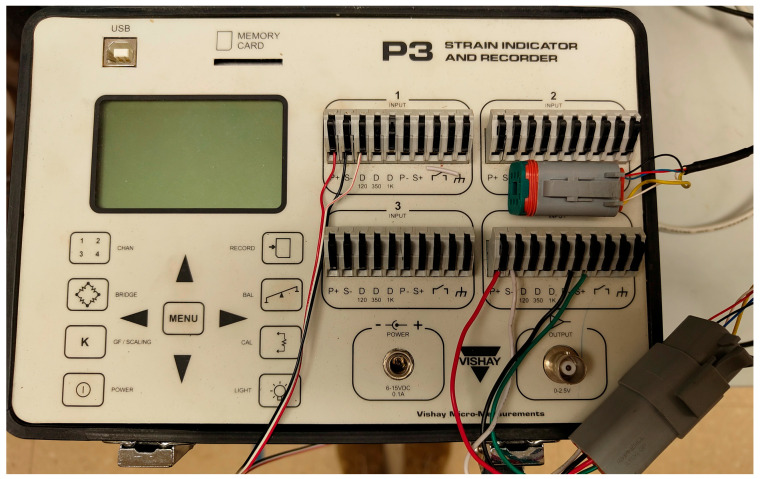
Quarter-bridge for the single strain gage mounted directly on the beam (channel 1) and full-bridge (channel 4) for the force-shunt strain transducer connections to P3.

Nominal strain measurements were acquired using a Micro-Measurements P3 strain indicator and recorder, manufactured by Vishay Precision group, Inc based in Wendall, NC, USA. As illustrated in Figure 4, electrical connections for the custom-built strain transducers were configured according to the wiring diagram in Figure 3, whereas the HBK SLB 700A was connected in accordance with the manufacturer’s published documentation. Amphenol Sine System’s AT Series connectors—specifically AT06-6S plugs and AT04-6P receptacles (original equipment manufacturer based in North America and Asia)—were used to ensure reliable signal transmission and minimize interference.

**Figure 5:**
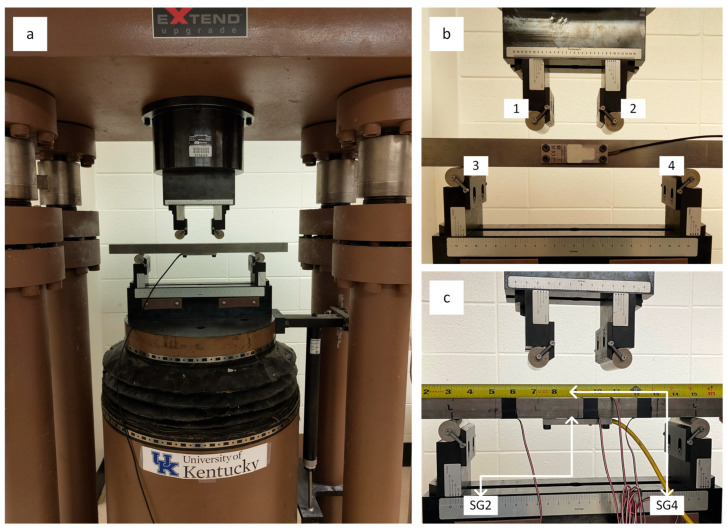
Experimental load testing system. (**a**) Load testing frame. (**b**) Four-point longitudinal bending load. (**c**) Four-point transverse bending load.

The same device was employed for all data acquisition, and although only its measurement accuracy and repeatability were used in the analysis, its accuracy and precision were independently confirmed through a four-point bending test. The specific method of applying axial load via the shunt was primarily selected due to the physical size of the transducers and their orientation relative to the four load points (labelled in Figure 5), which is akin to the intended installation position. The strain transducers were centered and mounted with M5 hex socket head screws to a 457 mm × 38.1 mm × 25.4 mm hot rolled steel beam (A36 steel beam shown in Figure 5b,c). The beam was centered and positioned perpendicular to the load points of the 4-point bend fixtures stationed on an MTS-815 dynamic and fatigue stiff testing system. The 4-point bending testing was conducted at a displacement-controlled rate of 0.762 mm/min, with the applied load recorded using Instron’s Bluehill 3 (legacy version 3) materials testing software. The strain (displacement) detected by both transducers was recorded with a Micro-Measurements P3 strain indicator and recorder and subsequently utilized to compute load (bending load). Equation (1) presents the relationship between bending load (*P*) and strain (*ε*) for a Young’s modulus of 200 GPa.

(1) $$P=\frac{2\;E\;\varepsilon \;I}{c\;(L_{2}-L_{1})}$$

where:

*L*_1_—represents the distance from the support to the nearest load point, and *L*_2_ the distance from the support to the furthest load point.

*c*—represents the distance from the neutral axis to the extreme edge.

*I*—represents the moment of inertia of the steel beam, and *E* is Young’s modulus of elasticity.

Additionally, to evaluate the response to bending for the transducers in force shunt, two Micro-Measurements EA-06-250BG-120 strain gages were secured on opposite sides of the steel beam with M-Bond AE-10 adhesive. To observe both tension and compression, Strain Gage 2 was attached directly to the beam underneath the force-shunt strain transducer, while Strain Gage 4 was attached to the side of the beam facing loading points 1 and 2. These gages were connected in quarter-bridge configurations.

**Figure 6:**
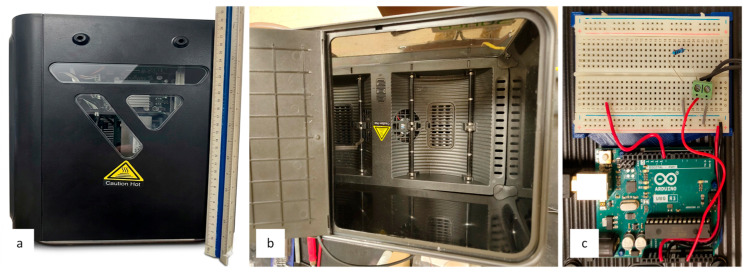
Environmental chamber. (**a**) Side view. (**b**) Top view. (**c**) Thermistor input to arduino.

The cyclical nature of the shuttle car loading and hauling process necessitated an investigation into temperature variability. A constant heating rate was selected based on the anticipated heating rate at the intended installation point of the strain transducer. To generate both uniform and non-uniform thermal gradients, ambient air temperature was controlled using an environmental chamber (Figure 6a,b) equipped with a PID controller. A precision thermistor was used to measure air temperature. Temperature determination employed a two-term approximation of the Steinhart–Hart equation, correlating the thermistor’s resistance to a reference resistance of 10 kΩ at 298.15 K. Heating and cooling cycles were subsequently controlled through data acquisition from the thermistor interfaced with an Arduino Uno R3 (Figure 6c). Two distinct protocols were used, in 5-min and 1.5-h increments, programmed in MATLAB (R2024b).

**Figure 7:**
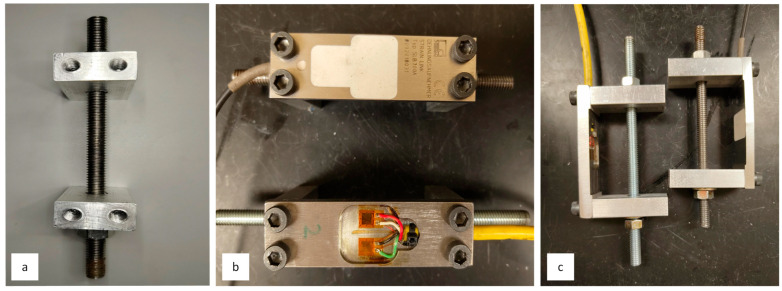
Loading of strain transducers in bending. (**a**) Top view of H-frame without transducers. (**b**) Top view of H-frame with transducers. (**c**) Side view of H-frame with transducers.

As shown in Figure 7, mechanical loading was applied through force shunt using H-frame brackets that include two arms measuring 50.8 mm × 25.4 mm × 12.7 mm, which are secured to the respective transducers with M5 hex socket head screws. By tightening bolts on opposite ends of the H-frame, a bending moment is induced at the outer edges of the strain transducer. This configuration ensures that strain gages positioned on the upper surface of the force-shunt transducer experience tensile strain (positive), while those on the underside are subjected to compressive strain (negative). The resulting loads from the force-shunt transducer are expressed in micro-strain and displayed via the P3 strain indicator operating on a balanced full Wheatstone bridge circuit.

The strain transducers subjected to force shunt remained exposed to their environmental conditions, and it is hypothesized that thermal stress compensation may vary under static and dynamic loading, particularly when non-uniform thermal gradients occur. For uniform thermal gradients, the two distinct transducer configurations underwent a two- to three-hour, three-stage heating cycle comprising heating, constant temperature, and cooling. Reflecting the cyclic nature of loading and hauling operations, the constant temperature (at 35 °C) and cooling stages were reduced to 5 min each and repeated four times. Deviations within ±5 micro-strain (−5 με to +5 με) from the induced loads were considered acceptable.

**Figure 8:**
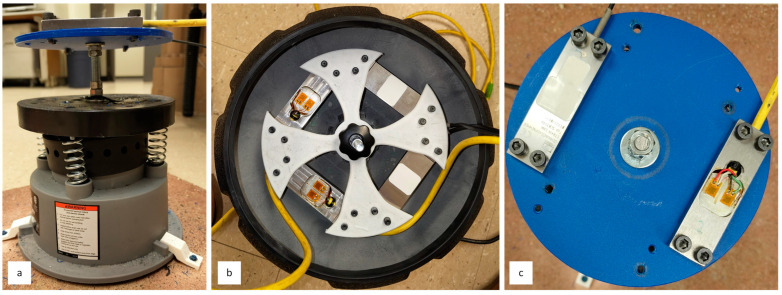
Vibration testing system. (**a**) Vibratory bowl (2.5 kg) (side view). (**b**) Original configuration for testing (side view). (**c**) Final configuration for testing (side view).

To replicate the intermittent nature of a typical stop-and-start loading cycle and uneven tramming conditions, vibration sensitivity was evaluated under both cyclic and continuous conditions. As depicted in Figure 8a, the transducers were mounted using M5 hex socket head screws to two different polyvinyl chloride fixtures, which were attached to a metal vibratory bowl powered by a 120 VAC, 0.5 Amp power supply. Fixture one, shown in Figure 8b, was 3D-printed, and Fixture two (Figure 8c) was produced by thermoforming. Although both mounting fixtures yielded similar results, the structural integrity of Fixture one was often compromised before the completion of a third test round and was thus replaced with Fixture two. Peak strain values were recorded across irregular vibration sequences (e.g., 20 min on, 2 min off; 10 min on, 5 min off; 5 min on) for durations up to 40 min. Additionally, considering the operational environment in which these strain transducers are intended to function, a continuous 6-h vibration test was performed without rebalancing to assess long-term stability.

## 4. Results and Discussion

This section describes in detail the response from two types of strain transducers subjected to vibration, mechanical, and thermal loads. Transducers were subjected to vibration tests, mechanical tests with varying torques and moments, and thermal tests at two distinct ambient air conditions for controlled 5-min and 1.5-h increments within the product specification parameters.

Additionally, the bending behavior of transducers was compared with that of strain gages at room temperature to confirm the accuracy of this experimental study and to illustrate the difference between force-flow and force-shunt load-transfer mechanisms. Ultimately, the findings of experimental tests are considered in light of the selection criteria for the operational conditions and the constraints associated with installation on shuttle cars.

### 4.1. Response to Vibration

Vibration excitation tests were conducted on the strain transducers at 59 Hz and 117 Hz, the frequencies measured with the vibratory bowl in operation. Although this experimental range is well within the manufacturer’s specifications of 10 Hz to 500 Hz, the SLB-700A models were the only transducers to show minor sensitivity to prolonged exposure to vibration. This was observed during the rebalancing of circuits at zero strain following the completion of cyclical testing (Table 1).

### 4.2. Bending Load Strain Measurements

**Figure 9:**
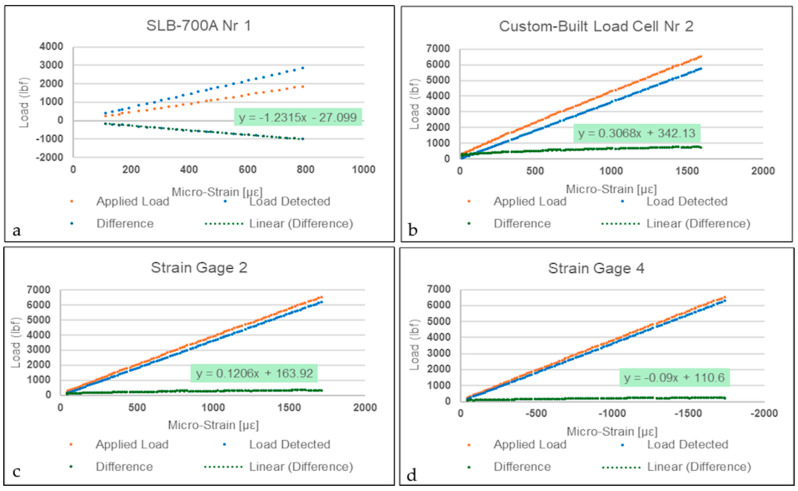
Result sample—(**c**) Measured response of strain gage 2 in tension and (**d**) strain gage 4 in compression in comparison with (**a**,**b**) strain transducers as a function of applied bending load.

As expected from strain gages in force shunt, the displacement detected by both transducers between loading points does not follow the applied load introduced to the beam. From the sampled results shown in Figure 9 it is also evident that the commercially available transducer is more sensitive to strain for increasing loads, above 100 με. To illustrate, only after an estimated applied load of 1 ton will the custom-built strain transducer reach 500 με (nominal measuring range of 0 to 500 με), while the SLB-700A achieves such at 0.5 ton. However, more remarkably, as shown in Figure 9, strain gages connected in a quarter-bridge circuit, intrinsically less sensitive than gages connected in full-bridge circuitry, deformed at nearly the same rate as the steel beam, which suggests that strain recorder equipment accurately reflects the behavior of the strain transducers.

### 4.3. Magnitude and Mechanisms of Thermal Dependence

The thermal dependence of full Wheatstone-bridge strain transducers in force shunt is affected by numerous factors originating from the environment in which they are installed, the transducer itself, or a combination of both. Apparent strain, in conjunction with transient effects, drift, and thermal zero shift, was evident from the thermo-mechanical response of the strain transducers and is discussed in the context of the experimental study.

#### 4.3.1. Apparent Strain and the Transient Effect

**Figure 10:**
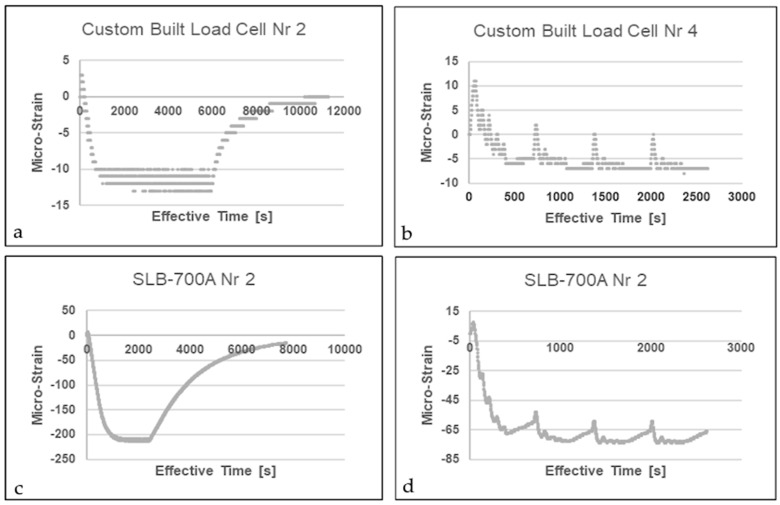
Result sample—induced thermal load at 35 °C (**b**,**d**) and 60 °C (**a**,**c**).

**Figure 11:**
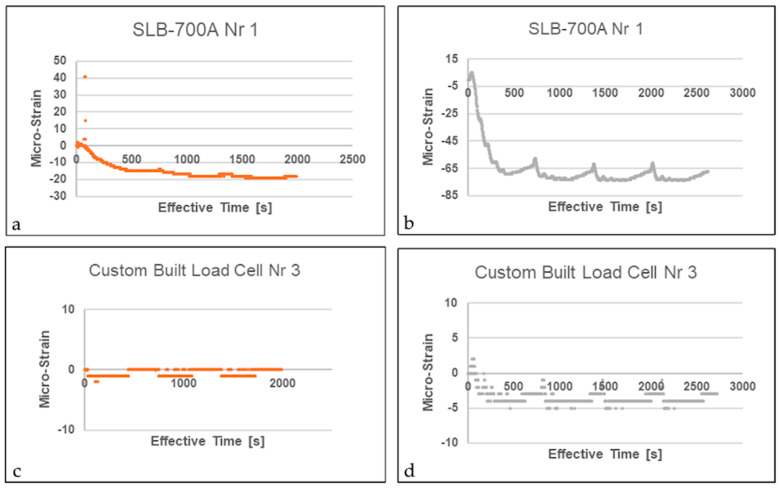
Result sample—induced thermal load at 35 °C for free-standing (**b**,**d**) and mounted (**a**,**c**) strain transducer.

Under a steady thermal gradient, the observed measurements ranged from −250 με in compression to +50 με in tension. While the HBK-SLB 700A strain transducer demonstrated a response reaching one-half of its nominal rated capacity, a notable phenomenon occurred at the start of several trials. Despite the Wheatstone bridge being pre-balanced for all mechanical loads, both transducers registered a tensile state at an ambient temperature of 60 °C. Since this behavior was observed exclusively during the initial heating phase and prior to the achievement of sustained thermal equilibrium, characterized by a momentary spike rather than a progressive lag or incremental buildup, adhesive softening, thermal drift (non-reversible zero-point change in strain gage), and handling-related damage are precluded as underlying mechanisms [14]. Realizing that strain gages are only compensated for a uniform temperature distribution provides insight into this phenomenon: the transducers experience localized heating until the temperature across the entire substrate is uniform. Thus, the temporary imbalance in the Wheatstone bridge can be attributed to a delayed change in the temperature gradient across the entire transducer substrate, stemming from some strain gages that are closer to the heat source than others. It is therefore apt to reconsider the applicability of the same gage factor for both static and dynamic measurements following this rapid change in electrical resistance. Nonetheless, as illustrated in the sampled results shown in Figure 10 and Figure 11, once heat is consistently circulated in the environmental chamber, the transient effect disappears, the Wheatstone bridge rebalances, and the mechanically induced load produces recordable strain.

Following the calibration and balancing of a strain gage system, any subsequent fluctuations in the ambient temperature of the installation site naturally induce a change in the transducer’s electrical resistance. Since the measurement instrumentation interprets any resistance variation as a physical deformation, these thermally induced fluctuations are recorded as apparent strain. Richards [16] established that a heating rate between 5.6 and 55.6 °C/s (10–100 °F/s) ensures that the error in measurements due to the transient effect is as substantial as those that arise from apparent strain. The transient effect is therefore hypothesized to intensify due to apparent strain that occurs during temperature changes; that is, the mismatch between the material strain gages situated on the transducer and the transducer substrate further exacerbates the transient effect, which cannot be accounted for by the full Wheatstone bridge circuit configuration. To a lesser extent, this phenomenon occurs at the end of cooling cycles as well, since mismatched expansion rates and temporary non-symmetrical heating, or rather cooling, on all four strain gages arise here as well.

#### 4.3.2. Drift

Drift describes the time-dependent variation in indicated strain when no mechanical stress is applied, and constant temperature is maintained at a steady state. During periods of continuous heating under a consistent thermal gradient, the transducers, which were configured with a gage factor of 2, demonstrated strain fluctuations limited to a 20 με range. It is noteworthy that the SLB-700A strain transducer displayed a substantially higher response even when the temperature only slightly exceeded ambient air levels. Nevertheless, across the three distinct phases of the heating protocol, the impact of cyclical thermal loading appears to stabilize quickly. As illustrated in Figure 10, the measurement range shows no significant deviation following the completion of the initial heating cycle. This finding is supported by the fact that temperature variations primarily influence the reference point, known as the zero point of measurements [14].

Drift in the absence of induced load via the mount and shunt is therefore repeatable and can be accounted for using analytical methods. The strain transducers are, however, expected to experience thermal as well as mechanical load simultaneously, which necessitates evaluation for thermal shift from a mechanically balanced state.

#### 4.3.3. Thermal Zero Shift

Consistent with the findings of Zhuang et al. [12], a zero-shift related to hysteresis, associated with mechanical or thermal cycling, was not observed for an anticipated operating temperature up to 60 °C. A difference in the indicated strain reading between an increasing and a decreasing thermal cycle under constant (including zero) mechanical strain conditions does, however, occur. This mechanism is demonstrated considering the indirect impact of thermal loading, which is evidenced by the strain transducers’ inability to revert to their baseline values. Both steady and, more significantly, irregular thermal gradients—as depicted in Figure 10 and Figure 11—hinder the restoration of a balanced state, regardless of whether a mechanical load was present at the start. Toggling between the maximum and minimum operating temperature thresholds at five-minute intervals enabled the quantification of the output range of erroneously indicated strain (listed in Table 2). The selection of the two loading settings was determined by the H-frame bracket’s moment and peak capacity limits. Analysis revealed that thermal and mechanical loads functioned independently across all four custom strain transducers, with no correlative link identified. Notably, however, the SLB-700A strain transducers exhibited a nearly linear correlation between mechanically induced load and strain under the peak recommended screw torque.

The difference in the indicated strain reading between an increasing and a decreasing mechanical strain under constant temperature conditions—often labeled as mechanical zero shift—was not investigated specifically, since induced mechanical load remains constant for the duration of each experiment. Yet, reaching a state of sustained heating, the two commercial transducers produced numerous data outliers once the H-frame brackets were installed. In contrast, the strain measurements displayed in Figure 11 appear more stable and reduced in magnitude for both strain transducers tested. To investigate how the mounting interface influences the thermo-mechanical response, force-shunt transducers were tested under three distinct fastening conditions: 16 N·m torque, 8 N·m torque, and secured by hand.

The thermo-mechanical response, documented in Table 3, was measured by subjecting all strain transducers to a 100 με load in compliance with the HBK SLB-700A mounting protocols. Under these conditions at 60 °C, the balanced circuits yielded peak ranges of 25 με and 55 με for the two units. These results demonstrate a strong correlation with the data previously established in Table 2. It is therefore apt to postulate that outliers are produced due to a temporary mechanical zero shift for all levels of mounting bolt tightening torque.

Short-duration transitions between heating and cooling phases were shown to induce a drift from the balanced state. Thus, to compensate for this drift, the circuits underwent rebalancing following the conclusion of the third three-stage heating cycle. This approach ensures that the prevailing and individual impacts of thermal loads are fully integrated into the preceding evaluations, reflecting the actual environmental conditions anticipated during operation. Given the permissible deviation threshold of ±5 με, the data from the initial balanced circuits suggest that bolt tightening torque does not significantly compromise the precision of the strain transducer under static loading conditions. Nevertheless, the rebalanced circuit data in Table 3 demonstrate that the SLB-700A strain transducers still maintain a slight vulnerability to prevailing environmental conditions.

## 5. Conclusions

The automation of loading and transport operations in an underground coal mine, particularly the operation of a flight chain, helps minimize work-related accidents and effectively reduces worker exposure to hazardous airborne pollutants. To advance knowledge and understanding of the requirements for these automated systems, an experimental study has been conducted on strain transducers in force shunt, with a specific focus on how unique environmental and installation factors affect the thermal accuracy of these readings. As per the cyclical nature and continuous use of wheel hubs on shuttle cars, a constant heating rate was selected based on the anticipated heating rate at the intended installation point of the strain transducer. Hence, the primary focus of the research was the environmental and anticipated operational factors unique to shuttle cars used in room-and-pillar mining operations, and the results quantify the thermo-mechanical response of full Wheatstone-bridge strain transducers in force shunt. Accordingly, the findings can serve as a reference for selection criteria in a similar application.

Thermal load induced at a non-uniform gradient remains constant within a range of 20 με after the first heating cycle, demonstrating consistent behavior. This stability relates to drift. Such variations primarily impact the reference zero point of measurements; however, because drift in the absence of induced load is repeatable, it can be effectively managed through analytical methods.

Rebalancing full Wheatstone bridge circuits at the conclusion of a three-stage thermal cycling protocol effectively mitigated drift from the initial balanced state. This process demonstrated that variations in mounting bolt torque had a negligible influence on the accuracy of strain measurements for the custom-built strain transducers. Vibration also proved to only affect the SLB 700-A strain transducers as a result of prolonged exposure. Nevertheless, by evaluating the performance of both transducers in force shunt, it was observed that the SLB-700A strain transducers exhibit a linear correlation between strain and mechanical load when operating at the boundaries of their temperature-compensated range. Conversely, the four custom-engineered strain transducers demonstrated a distinct lack of this relationship, instead showing that thermal and mechanical loads functioned independently of one another. This independence is a critical finding, as it confirms that the custom designs are successfully insulated from the parasitic environmental effects they were built to withstand. However, discrepancies in strain readings under uniform thermal conditions suggest that the interaction between the transducer and the H-frame bracket material plays a significant role in data output. It is suggested that these anomalies result from a temporary mechanical zero shift that occurs irrespective of how tightly the mounting bolts are torqued.

Furthermore, the operational stability of these strain transducers is hindered by the fact that they do not consistently return to their original balanced state after the initial load is applied, due to lingering thermal dependence. This phenomenon often results in the transducers experiencing a residual tensile state following a 1.5-h sustained cooling period unless the Wheatstone bridge circuits are frequently recalibrated. Ultimately, the research indicates that the custom-built force-shunt strain transducers are an effective means for mapping the profile and location of coal in shuttle cars, provided that the systems are subjected to continuous and cyclic rebalancing to maintain accuracy.

Since strain transducers are expected to operate outside steady-state conditions, they remain exposed to conditions in which the temperature is not uniformly distributed throughout the transducer substrate, and transient effects, in conjunction with apparent strain, are expected to persist. To address this phenomenon, the indicated strain must be known for each heat cycle and subtracted from the true strain. Prior to applying analytical methods to account for drift and determining the requirements of means to correct for the erroneous strain reading inflected by the transient effect in conjunction with apparent strain, the practicality of continuously re-balancing circuits requires further investigation.

## Figures and Tables

**Table 1:** Maximum and minimum strain under cyclical and continuous vibration loading.

	Custom Built Transducers		SLB-700A Transducers	
	Initial Balanced Circuit (0 με)	Rebalanced Circuit (0 με)	Initial Balanced Circuit (0 με)	Rebalanced Circuit (0 με)
**Cyclic**	+1 με to −11 με	+3 με to −3 με	+2 με to 0 με	+2 με to 0 με
**Continuous** **(6-h)**	+4 με to −4 με		+2 με to −14 με	

**Table 2:** Impact of induced mechanical load at 35 °C and 60 °C at 16 N·m tightening torque.

Custom Built Transducer		SLB-700A Transducer		
35 °C & 60 °C			35 °C	60 °C
Unloaded	+10 με to −10 με	Unloaded	+5 με to −20 με	+5 με to −70 με
Loaded (200 με)	+210 με to +190 με	Loaded (50 με)	−45 με to −75 με	−45 με to −110 με
Loaded (300 με)	+310 με to +290 με	Loaded (100 με)	−95 με to −130 με	−95 με to −155 με

**Table 3:** Impact of bolt-torque-generated loading during installation at 60 °C.

	Custom Built Transducer		SLB-700A Transducer	
	Initial Balanced Circuit (100 με)	Re-Balanced Circuit	Initial Balanced Circuit (100 με)	Re-Balanced Circuit
**Secured by Hand**	+115 με to +90 με	+5 με to −3 με	−95 με to −160 με	+2 με to −6 με
**8 N·m**	+110 με to +85 με	+5 με to −6 με	−95 με to −160 με	+2 με to −14 με
**16 N·m**	+110 με to +90 με	+3 με to −4 με	−95 με to −150 με	+1 με to −8 με

## Data Availability

Data is available upon request. A large amount of data was collected; some of it is presented in graphs and figures included in this paper. There are no restrictions on the collected data.
